# The Association of Severe Coronary Tortuosity and Non-Obstructive Coronary Artery Disease

**DOI:** 10.3390/medicina59091619

**Published:** 2023-09-07

**Authors:** Petra Zebic Mihic, Sandra Saric, Ines Bilic Curcic, Ivan Mihaljevic, Iva Juric

**Affiliations:** 1 Department of Cardiovascular Diseases, University Hospital Center Osijek, 31000 Osijek, Croatia; 2 Faculty of Medicine Osijek, Josip Juraj Strossmayer University of Osijek, 31000 Osijek, Croatia; 3 Department of Endocrinology, University Hospital Center Osijek, 31000 Osijek, Croatia; 4 Department of Nuclear Medicine, University Hospital Center Osijek, 31000 Osijek, Croatia

**Keywords:** non-obstructive coronary artery disease, women, coronary tortuosity, left coronary dominance, myocardial ischemia

## Abstract

*Background and Objectives:* There is an increasing interest in the coronary tortuosity as a novel pathophysiological mechanism of ischemia in coronary artery disease without significant obstruction, but there are a lack of studies to confirm this relationship in the clinical setting. The aim of our study was to evaluate the association of severe coronary tortuosity and the potential role of coronary blood supply dominance in the appearance of myocardial ischemia in patients with non-obstructive coronary artery disease (non-CAD), compared to patients with obstructive coronary artery disease (CAD). *Materials and Methods:* The study enrolled 131 participants (71 male and 60 female), recruited among patients referred to cardiologists due to angina symptoms with ischemic alterations established by cardiac stress tests, as well as those admitted to the hospital for acute coronary syndrome. *Results:* Mean age of recruited patients was 61.6 (±10.1) years. According to the coronary angiography, they were divided into two groups: non-obstructive and obstructive CAD (77 and 54, respectively). There were significantly more women (61% vs. 24%, *p* < 0.001) in the non-CAD group. Both tortuous coronary arteries (50.6% vs. 14.8%, *p* < 0.001) and left coronary dominance (37.7% vs. 16.7%, *p* = 0.006) were more frequent in the non-CAD group compared to the CAD group. Female sex (OR = 17.516, *p* = 0.001), tortuous coronary arteries (OR = 7.962, *p* = 0.006) and left dominance of blood supply were significant predictors for non-CAD. *Conclusions:* Non-obstructive CAD is common among patients, especially women, who are referred for coronary angiography. Severe coronary artery tortuosity is the strongest independent predictor of non-obstructive CAD, followed by female gender and left coronary dominance.

## 1. Introduction

Despite improvements in prevention, diagnosis and treatment, ischemic heart disease remains the leading cause of morbidity and mortality in developed countries.

A clinical entity characterized by symptoms and signs of myocardial ischemia without significant angiography-proven coronary artery obstruction has been recently recognized as the common cause of ischemic heart disease, but is not yet sufficiently investigated. Non-obstructive coronary artery disease is a burden on the healthcare system and on patients, which affects quality of life in different ways. Recurrent angina symptoms without a specific pathological substrate disrupt the physical and psychological condition of patients, causing repeated hospitalizations and coronary angiographies. Studies have shown that more than 50% of patients with angina symptoms had no obstructions on coronary angiography although they had proven myocardial ischemia [1,2,3]. It has been shown that those patients have an increased risk for major cardiovascular events including cardiovascular death, heart failure, myocardial infarction, stroke and all-cause mortality [4,5]. Gender differences play an important role in patients with non-obstructive coronary artery disease, as it was proposed that ischemic signs and symptoms have different pathophysiological mechanisms in women and men [6].

Coronary artery tortuosity is a commonly detected coronary angiography finding, but is rarely reported by cardiologists [4]. The probable reason for this attitude is that despite growing interest in non-obstructive coronary artery disease, there is a lack of evidence about the association between coronary artery tortuosity and development of myocardial ischemia without significant coronary obstruction. Some previous studies suggest that tortuosity of coronary arteries may cause the reduction in distal perfusion pressure and coronary flow that leads to the myocardial ischemia in regions supplied by tortuous arteries [7]. This was shown through computational simulations and explained by fluid mechanics, but there are not enough studies to support this relationship in the clinical setting [8,9]. A recent retrospective study, which included 57 patients with angina and non-obstructive coronary artery disease, showed the correlation between coronary tortuosity and myocardial ischemia, but there is a need for further research on a bigger population [4]. The role of coronary tortuosity in the development of acute coronary syndrome has not been previously investigated. There is a higher prevalence of coronary tortuosity among women, but the mechanism of this correlation is not explained [4]. Hypertension and advanced age are also found to be risk factors for coronary tortuosity, probably through their long-standing impact on the heart structure and function [4].

Therefore, further investigation of the angiographic characteristics of coronary circulation beyond the degree of coronary obstruction was needed. The aim of this study was to evaluate the association of severe coronary tortuosity and the potential role of coronary blood supply dominance in the appearance of myocardial ischemia in patients with non-obstructive compared to obstructive CAD. We have also assessed the role of established cardiovascular risk factors in non-obstructive CAD.

## 2. Materials and Methods

### 2.1. Patients and Procedures

This was a single-center cross-sectional study conducted at the University Hospital Center Osijek during the period of 12 months from December 2021 to December 2022. The study was approved by the Institutional Ethics Committee. Written informed consent was obtained from all participants before the engagement in the study.

A total of 131 participants (71 male and 60 female) were recruited in the study, which enrolled patients over 18 years old with suspected coronary artery disease who underwent outpatient cardiac examination and those admitted to the hospital in the acute setting. Outpatient participants were recruited if they had angina symptoms and if they underwent an exercise stress test or myocardial perfusion scintigraphy, demonstrating ischemic alterations. Acute patients were admitted to the hospital with the diagnosis of acute coronary syndrome, including myocardial infarction with or without ST-segment elevation and unstable angina. All patients underwent coronary angiography based on the clinical indications. After diagnostic coronary angiography, if there was an indication, depending on the decision of the revascularization team and the operator with the consent of the subject, a percutaneous coronary intervention was performed. All subjects were treated in accordance with the current guidelines of the European Society of Cardiology [10,11].

Patients were interviewed to obtain medical history, demographic characteristics and behavioral habits. Both interviews and a review of medical records were used to determine the presence of cardiovascular risk factors. Clinical data including age, gender, family history and history of comorbidities, such as hypertension, diabetes mellitus, hyperlipidemia, and history of smoking and alcohol consumption were collected and compared. Following a detailed clinical examination performed by the cardiologist, patients underwent the echocardiographic examination with an evaluation of systolic and diastolic function and valvular disease. Laboratory testing of the lipid profile and homocysteine level was also performed.

All patients were subjected to invasive coronary angiography with an assessment of the degree of coronary obstruction, severity of coronary tortuosity and type of coronary blood supply. According to the results, they were divided in two groups—the group with non-obstructive coronary artery disease (without obstruction or with obstruction of less than 50%) and the group with obstructive coronary artery disease (obstruction ≥ 50%) [1]. All coronary angiograms were performed by conventional methods via radial or, alternatively, femoral approach and were filmed at 15 frames per second by the Philips Azurion 7 M20 radiographic unit. Angiograms were analyzed offline by two expert cardiologists who were both blinded to the study protocol.

**Figure 1:**
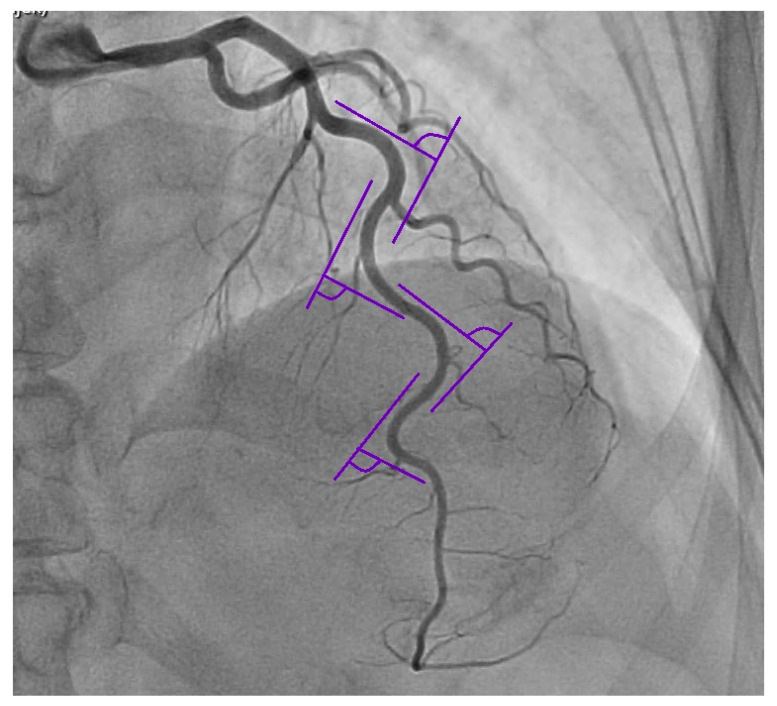
Representation of severe tortuosity of the coronary artery with bend angles measured according to our criteria.

Angiographic analysis was performed in diastole and in systole from two-dimensional angiographic recordings. The left anterior descending coronary artery (LAD), the left circumflex coronary artery (LCX) and the right coronary artery (RCA) were observed by various angulations: RCA the 30-degree right anterior oblique view and the 30-degree left anterior oblique view; LAD the 30-degree right anterior oblique view with 60-degree cranial and the 30-degree left anterior oblique view with 60-degree cranial; LCX the 30-degree left anterior oblique view with 30 degrees of caudal angulation and at the 30-degree right anterior oblique view with 30 degrees of caudal angulation. The visually assessed luminal coronary obstructions of ≥50% in at least one main coronary artery were considered as CAD. We included patients with severe tortuosity, which was identified by the presence of ≥3 consecutive bends (presence of ≥45° change in vessel direction) along the main trunk (measured from their emergence in the aortic root or left main coronary artery to their ending, with exclusion of side branches) of at least one coronary artery (LAD, LCX, RCA) present in both systole and diastole [12], which is presented in Figure 1. An assessment of the coronary artery blood supply dominance was also performed. According to the arterial supply of the posterior descending artery (PDA) the patients were divided in three groups: (1) the right dominance group with PDA originating from the right coronary artery; (2) the left dominance group with PDA arising from the left circumflex artery; (3) balanced circulation group with PDA supplied from both the RCA and LCX [13].

Patients were excluded from the study if any of the following criteria were present: heart failure with impaired systolic function, diastolic dysfunction greater than impaired relaxation, pulmonary hypertension, congenital disease, valvular heart disease, myocardial bridging, anemia, patients with malignant disease or other chronic diseases that would cause symptoms similar to angina (chronic obstructive pulmonary disease, musculoskeletal diseases and psychological disorders).

### 2.2. Statistical Analysis

Statistical analysis was performed using the statistical software package Statistica v.12 (StatSoft, Inc.,Tulsa, OK, USA) and MedCalc v.20 (MedCalc Software Ltd., Ostend, Belgium). Categorical variables were presented as numbers and percentages (%) and for comparisons between groups chi-square test was used. Continuous variables were presented as medians and interquartile range (IQR) and comparisons between groups were done using the Mann–Whitney test because the distribution of most variables was not normal (tested by the Kolmogorov–Smirnov test). The association of variables (continuous and categorical) with non-obstructive coronary artery disease was determined using logistic regression analysis. *p* < 0.05 was used as statistically significant for all performed analyses.

## 3. Results

The study recruited 131 patients (71 male and 60 female) referred to our clinic due to suspected coronary artery disease. After the coronary angiography, patients were divided into two groups—the group with non-obstructive coronary artery disease (non-CAD), including 77 patients (58.8%) and the group with obstructive coronary artery disease (CAD) including 54 patients (41.2%). The study participants’ demographics, vitals and personal history data are presented in Table 1. Mean age of recruited patients was 61.6 (±10.1) years. The groups were comparable for age (63 vs. 62 years, *p* = NS (non-significant)) and BMI (27.77 vs. 29.31 kgm^−2^, *p* = NS).

As seen in Table 1, there were significantly more women (61% vs. 24%, χ^2^ = 17.3, *p* < 0.001) and non-smokers in the non-CAD group (76.6% vs. 23.4%, χ^2^ = 4.5, *p* = 0.034). Alcohol consumption, diabetes, hypertension, hyperlipidemia and positive family history were present in comparable proportions between the subgroups (*p* > 0.05 for all). Referral diagnosis for non-CAD and CAD was significantly different, with a significantly higher proportion of patients with chronic coronary syndrome (71.4%) in the non-CAD group and predominant acute coronary syndrome in the CAD group (74.1%) (χ^2^ = 77.6, *p* < 0.001, Table 1).

Lipid profiles are presented in Table 2. As seen in Table 2 homocysteine and triglycerides were significantly lower (1.04 vs. 1.54 mmol/L, *p* < 0.001), and HDL (1.26 vs. 1.08 mmol/L, *p* = 0.019) and HDL/cholesterol (27% vs. 23%, *p* < 0.001) significantly higher in the non-CAD group compared to the CAD group. Levels of cholesterol and LDL were comparable between groups (*p* > 0.05 for both) (Table 2).

The tortuosity of coronary arteries and the type of blood supply are presented in Table 3. Significantly more tortuous coronary arteries were found in the non-CAD group (50.6% vs. 14.8%, χ^2^ = 17.6, *p* < 0.001). This was especially the case for tortuous LCX (35.1% vs. 9.3%, χ^2^ = 11.4, *p* < 0.001), but also for LAD (27.3% vs. 11.1%, χ^2^ = 5.03, *p* = 0.025) and RCA (11.7% vs. 0.0%, χ^2^ = 6.73, *p* = 0.010) (Table 3). Coronary artery blood supply was predominantly right (70.4% vs. 59.7%) in both subgroups but there was significantly more left coronary dominance in the group of patients with non-CAD (37.7% vs. 16.7%, χ^2^ = 10.3, *p* = 0.006, Table 3).

**Figure 2:**
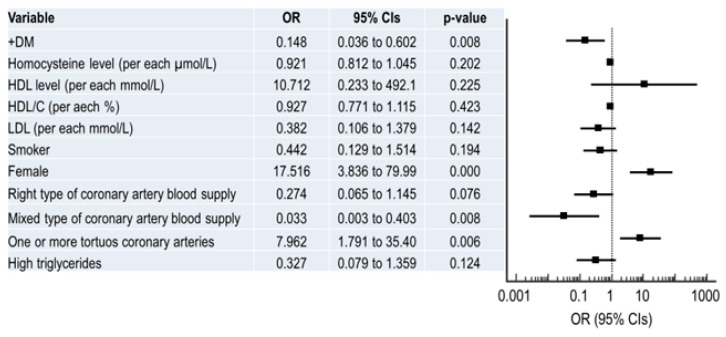
Results of the multivariate logistic regression for non-obstructive CAD; OR—odds ratio, CI—confidence interval.

Differences in demographics, history, lipid profiles and data from coronary angiography were assessed using multivariate logistic regression for predictors of non-CAD vs. CAD. The results of the multivariate logistic regression analysis are presented in Figure 2. Significant predictors for non-CAD were one/more tortuous coronary arteries (OR = 7.962, 95% CI 1.791 to 35.40, *p* = 0.006) and female sex (OR = 17.516, 95% CI 3.836 to 79.99, *p* = 0.001) (R^2^ = 0.69, *p* < 0.001 for the model; Figure 2). Left dominance of coronary artery blood supply was a significant predictor of non-CAD when compared to balanced type (*p* = 0.008) and borderline when compared to right dominance (*p* = 0.076). Not having diabetes (OR = 0.148, 95% CI 0.036 to 0.602, *p* = 0.008) was also found to be a significant predictor of non-CAD (Figure 2). The model presented an accuracy of 85.4% for non-CAD diagnosis compared to CAD, with an AUC of 0.932 (95% CI, 0.874 to 0.969). As can be seen in Figure 2, the most significant independent predictor for non-CAD, with a very narrow 95% Cis, was the presence of a tortuous coronary artery, increasing the odds for non-CAD eight times, followed by being a member of the female sex (increasing the odds 17.5 times).

## 4. Discussion

There has been a growing interest in the pathophysiological mechanisms and clinical outcomes of non-obstructive coronary artery disease since it was recognized as a significant cause of ischemic heart disease and major cardiovascular events, especially in the female population [1,14,15]. Our study examined the occurrence of non-obstructive CAD in the group of patients referred to coronary angiography for suspected coronary artery disease and the association of coronary tortuosity and the existence of non-obstructive CAD. We also analyzed the relationship between coronary blood supply dominance type and non-obstructive CAD occurrence and assessed cardiovascular risk factors in relation to patients with obstructive CAD.

Our study showed that almost 60% of the patients with signs of myocardial ischemia in acute or chronic settings were diagnosed with non-obstructive coronary artery disease. More than 70% of the patients referred for elective coronary angiography indicated through symptoms and ischemic alterations found on an exercise stress test or myocardial perfusion scintigraphy, that they had non-obstructive coronary arteries, which is consistent with previous research and shows the importance of this clinical entity [1,2,3,4,16]. As stated in earlier studies, our research also showed that there was a significantly higher prevalence of non-obstructive CAD among women [1,14,15,16]. Almost 80% of women recruited in this study presented as CAD without significant obstruction of coronary arteries.

The role of established cardiovascular risk factors in the development of non-obstructive CAD is not yet clarified. Although it is proposed that age, diabetes, hypertension and hyperlipidemia have an impact on microvascular dysfunction, most studies observed a lower incidence of traditional risk factors in the population with non-obstructive coronary artery disease [3,14,17]. Our study showed comparable proportions of hypertension, hyperlipidemia, family history and alcohol consumption between the subgroups, but significantly more smokers in the subgroup with obstructive CAD. Since these are the established risk factors for CAD, comparable results in the subgroups may indicate that there is some impact of these risk factors on the development of non-obstructive CAD, especially of hypertension which was detected in almost 80% of our patients with non-obstructive CAD, but further investigations are needed to determine this dependence. In our study, diabetes was less present in non-CAD patients. This did not reach significance, but showed as an independent risk factor for CAD in a multivariate analysis. Similar results for diabetes were found in previous studies [2,3,5,14]. According to the laboratory testing at the time of hospital admission, we observed a more favorable lipid profile in the group of patients without significant coronary artery obstruction, including significantly higher levels of HDL cholesterol and lower levels of triglycerides, which supports the fact that hyperlipidemia has a more significant role in the obstructive CAD. Homocysteine levels, which are considered to be a risk factor for CAD, were also analyzed. Significantly higher levels of homocysteine in the group with obstructive CAD were observed, which is consistent with previous studies and confirms the role of homocysteine as the risk factor for obstructive CAD, but not for non-obstructive CAD [18,19].

A frequently observed but often ignored coronary angiography finding is the tortuosity of the coronary arteries. This insufficiently explored entity may play a significant role in the development of ischemic signs and symptoms in patients without significant coronary obstruction. Several previous studies proposed coronary tortuosity as a potential cause of ischemia in non-obstructive CAD, but there are a lack of studies to confirm this relationship in the clinical setting [4,20,21]. A possible mechanism for developing ischemia is a reduction in distal perfusion pressure and coronary flow in the myocardial regions supplied by tortuous coronary arteries. This may be a result of an increased friction, leading to shear stress and a pronounced centrifugal effect appearing in tortuous arteries [7]. The main determinants of these phenomena are the number of bends and the extent of the bend angles [4]. Li et al. made a computational simulation of pressure dynamics in tortuous coronary arteries and showed a significant decrease of coronary pressure distal to the tortuous segment, related to the severity degree of tortuosity [8], which was confirmed in later studies [9].

Our study showed that 50% of patients with non-obstructive CAD and ischemic alterations had a severe degree of coronary tortuosity. The occurrence of tortuosity was significantly higher compared to patients with obstructive CAD. This finding suggests this entity could be a potential cause of myocardial ischemia in patients with non-CAD. Most cases of the tortuous coronary artery were found in LCX, followed by LAD and RCA, but with a significantly higher appearance in the non-CAD group than in the CAD group for all three blood vessels. A higher occurrence of tortuous LCX, followed by LAD was found in the previous studies and can be related to the higher myocardial wall thickness observed in this region, leading to the genesis of tortuosity [22,23,24,25]. A recently published study by Estrada et al. showed that the number of coronary bend angles in systole may be an indicator of increased risk of myocardial ischemia and found more pronounced coronary tortuosity (according to the number and magnitude of bend angles) in the LCX associated with the greatest frequency of ischemia in that territory [4].

Although it is known that left coronary dominance is related to a worse prognosis in obstructive CAD, the impact of the coronary blood supply dominance on ischemic alterations in non-obstructive CAD is not clearly understood [13]. Previous studies suggested a higher prevalence of non-obstructive CAD in women with left dominance [26]. It is also stated that left dominance may have a prognostic role in angina-related hospitalizations in women with non-obstructive CAD [10]. As in earlier investigations, right coronary supply also predominates in both subgroups in our study [16]. We also found a significantly higher proportion of left coronary dominance in the group with non-obstructive CAD. In contrary with previous studies, we did not observe significant sex-specific differences according to the coronary dominance in the group of non-obstructive CAD. The impact of the left dominance on the development of ischemia is explained by the less developed right coronary artery in this setting, which could cause regional ischemia in the area supplied by this artery, regardless of the absence of significant artery obstruction [26]. We propose that in the left dominance of coronary supply, coronary tortuosity, which is most frequently observed in a circumflex artery, may cause regional ischemia in the area supplied by the posterior descending artery, as well as in the area supplied by the circumflex artery itself, leading to ischemic symptoms and signs in the population affected by this anatomical variation. Further investigations with matching types of coronary supply and exact regions of ischemia are needed to clarify this relationship.

Previous studies showed a higher prevalence of severe coronary tortuosity among women [6,27]. In addition, it seems that coronary tortuosity may be positively related to age, hypertension and impaired left ventricular relaxation [22,25,28,29], but negatively related to enlargement of the heart [28]. Groves et al. did not find a correlation between coronary artery tortuosity and hypertension, hyperlipidemia, diabetes, family history, smoking and older age, but demonstrated a significantly lower incidence of significant CAD in patients with coronary tortuosity and a higher incidence of tortuosity among women, which is consistent with our study [30]. Structural and functional changes in the heart which arise because of aging and long-standing hypertension may lead to the development of coronary tortuosity [6]. Coronary tortuosity represents an adaptive mechanism of coronary arteries to the conditions of chronic exposure to high flow and high parietal stress, which is augmented in hypertensive patients. Tortuosity formation is also affected by elastin decrease in the arterial wall occurring in advanced age [21]. These facts indicate a possible relationship of aging and hypertension with coronary tortuosity. Contrary to previous studies, in our study, a significant relationship between coronary tortuosity and advanced age and hypertension was absent, although a weak association was present. This discrepancy may be caused by a relatively small age range and an unspecified degree and duration of hypertension.

The correlation between coronary tortuosity and atherosclerosis of coronary arteries remains disputable. Some studies described that coronary tortuosity is linked to subclinical atherosclerosis and promotes atherosclerotic changes due to shear stress [7,31,32]. Other studies showed significantly fewer atherosclerotic changes in tortuous coronary arteries and proposed that the higher sheer stress observed in tortuous arteries may present protection against atherosclerosis [9,33]. The fact that we observed a significantly lower occurrence of obstructive coronary artery disease in patients with tortuous coronary arteries indicates that tortuosity of coronary arteries may act as the protective mechanism against atherosclerosis, which was also stated in previous studies [34].

Besides changes in epicardial coronary arteries, microvascular dysfunction takes an important role in the development of myocardial ischemia in non-obstructive CAD. It is known that over 90% of the flow resistance in coronary circulation is generated by the coronary microvasculature system [35]. Except for known invasive methods for assessment of the coronary microcirculation, there are some non-invasive techniques proposed for the early detection of coronary microvascular dysfunction and ischemia, such as myocardial contrast stress echocardiography or computational electrocardiogram and vectorcardiogram analysis [36,37,38,39]. Investigation of multifactorial etiology of non-obstructive CAD could be of interest, because the tortuosity of epicardial coronary arteries in combination with microvascular dysfunction could worsen the course of the disease. Integrated evaluation of coronary tortuosity and changes in microvasculature could represent a new approach in early detection of non-obstructive CAD.

This study has several strengths. This is an interesting topic that is not sufficiently investigated and this study brings new insights about the significant role of coronary tortuosity and left coronary dominance in non-obstructive CAD. These results were obtained from a comparison to obstructive CAD, which was not performed in previous studies. Our unbiased population sample provides real clinical data. The limitation of this study is the relatively small group of patients from a single center. Further multicentric studies with prospective follow-up, including more patients, are needed. Determination of the tortuosity index or performing coronary functional testing would also contribute to the plausibility of the results.

## 5. Conclusions

There is a high prevalence of non-obstructive coronary artery disease among the patients referred for coronary angiography indicated by angina symptoms and ischemic alterations, especially among women. Tortuosity of coronary arteries, as well as left dominance blood supply, are more common in patients with non-obstructive CAD compared to patients with obstructive CAD. Severe tortuosity of coronary arteries is the strong independent predictor for non-obstructive coronary artery disease, followed by female sex and left coronary dominance.

## Figures and Tables

**Table 1:** Demographics, vitals and personal history data according to the groups (N = 131).

Variable	Group		Statistics
	Non-Obstructive CAD (N = 77)	Obstructive CAD (N = 54)	
Female sex; N (%)	47 (61)	13 (24)	χ^2^ = 17.3, *p* < 0.001
Age, years; M (IQR)	63 (55–69)	62 (57–68)	0.929 *
Height, m; M (IQR)	1.67 (1.62–1.77)	1.71 (1.65–1.78)	0.184 *
Weight, kg; M (IQR)	80 (68–93.25)	85 (76–95)	0.169 *
BMI, kgm^−2^; M (IQR)	27.77 (25.95–31.53)	29.31 (26.51–31.77)	0.285 *
Smoking; N (%) Non-smokers Smokers	59 (76.6) 18 (23.4)	32 (59.3) 22 (40.7)	χ^2^ = 4.5, *p* = 0.034
History; N (%) Alcohol DM HTA HLP FH	4 (5.2) 17 (22.1) 61 (79.2) 32 (41.6) 38 (49.4)	3 (5.6) 20 (37.0) 41 (75.9) 29 (53.7) 21 (38.9)	χ^2^ = 0.01, *p* = 0.928 χ^2^ = 3.5, *p* = 0.062 χ^2^ = 0.2, *p* = 0.656 χ^2^ = 1.9, *p* = 0.172 χ^2^ = 1.4, *p* = 0.238
Dg of referral; N (%) CCS ACS	55 (71.4) 22 (28.6)	14 (25.9) 40 (74.1)	χ^2^ = 26.2, *p* < 0.001

Legend: * Mann–Whitney U-test; M—median, IQR—interquartile range, χ^2^—chi-square, DM—diabetes, HTA—arterial hypertension, HLP—hyperlipidemia, FH—family history, Dg—diagnosis, CCS—chronic coronary syndrome, ACS—acute coronary syndrome, CAD—coronary artery disease.

**Table 2:** Lipid profiles and homocysteine levels according to the groups (N = 131).

Variable	Group		Statistics
	Non-Obstructive CAD (N = 77)	Obstructive CAD (N = 54)	
Cholesterol level, mmol/L; M (IQR) Increased; N (%)	4.65 (3.63–5.43) 27 (35.1)	4.66 (3.92–5.75) 22 (40.7)	*p* = 0.310 * χ^2^ = 0.43, *p* = 0.510
HDL level, mmol/L; M (IQR) Low; N (%)	1.26 (1.02–1.49) 17 (22.1)	1.08 (0.96–1.34) 20 (37.0)	*p* = 0.019 * χ^2^ = 3.48, *p* = 0.062
HDL/C, %; M (IQR) Low; N (%)	27 (23–34.25) 6 (7.8)	23 (20–27) 18 (33.3)	*p* < 0.001 * χ^2^ = 13.73, *p* < 0.001
LDL level, mmol/L; M (IQR) Increased; N (%)	2.88 (2.08–3.64) 75 (97.4)	3.19 (2.57–3.83) 53 (98.1)	*p* = 0.057 * χ^2^ = 0.08, *p* = 0.780
Triglycerides level, mmol/L; M (IQR) Increased; N (%)	1.04 (0.67–1.42) 15 (19.5)	1.54 (1.29–1.98) 21 (38.9)	*p* < 0.001 * χ^2^ = 5.95, *p* = 0.015
Homocysteine level; M (IQR) Increased; N (%)	10 (8.75–12.0) 10 (13.0)	11 (10.0–14.0) 10 (18.5)	*p* = 0.014 * χ^2^ = 0.75, *p* = 0.388

Legend: * Mann–Whitney U-test; M—median, IQR—interquartile range, χ^2^—chi-square, HDL—high-density lipoprotein, C—cholesterol, LDL—low-density lipoprotein, CAD—coronary artery disease.

**Table 3:** Tortuosity of coronary arteries and type of blood supply according to the groups (N = 131).

	**Group**		**Statistics**
**Variable**	**Non-Obstructive CAD (N = 77)**	**Obstructive CAD (N = 54)**	
Tortuous coronary artery; N (%) LCX LAD RCA	39 (50.6) 27 (35.1) 21 (27.3) 9 (11.7)	8 (14.8) 5 (9.3) 6 (11.1) 0 (0.0)	χ^2^ = 17.6, *p* < 0.001 χ^2^ = 11.4, *p* < 0.001 χ^2^ = 5.03, *p* = 0.025 χ^2^ = 6.73, *p* = 0.010
Type of coronary artery blood supply; N (%) Left Right Mixed	29 (37.7) 46 (59.7) 2 (2.6)	9 (16.7) 38 (70.4) 7 (13.0)	χ^2^ = 10.3, *p* = 0.006

Legend: χ^2^—chi-square, LCX—left circumflex coronary artery, LAD—left anterior descending coronary artery, RCA—right coronary artery, CAD—coronary artery disease.

## Data Availability

The data presented in this study are available on request from the corresponding author. The data are not publicly available due to ethical reasons.
